# Supplementary material for: Flexible and stretchable metal oxide nanofiber networks for multimodal and monolithically integrated wearable electronics
Source: Nat Commun. 2020 May 15;11:2405. doi: 10.1038/s41467-020-16268-8 (PMC7229221; doi:10.1038/s41467-020-16268-8)
Supplement: Supplementary file 1 — Supplementary Information [file 41467_2020_16268_MOESM1_ESM.pdf]

Supplementary Information

Flexible and Stretchable Metal Oxide Nanofiber Networks for Multimodal and Monolithically Integrated Wearable Electronics

Binghao Wang,[1,⊥] Anish Thukral,[2,⊥] Zhaoqian Xie,[3,4] Limei Liu,[5] Xinan Zhang,[1,6] Wei Huang[1], Xinge Yu,[3*] Cunjiang Yu,[2*] Tobin J Marks,[1,5*] and Antonio Facchetti[1,7*]

[1]Department of Chemistry and the Materials Research Center, Northwestern University, 2145 Sheridan Road, Evanston, IL 60208, USA

[2]Department of Mechanical Engineering, University of Houston, 4726 Calhoun Rd, Houston, TX, 77004 USA

[3]Department of Biomedical Engineering, City University of Hong Kong, 83 Tat Chee Avenue, Kowloon Tong, Hong Kong, China

[4]State Key Laboratory of Structural Analysis for Industrial Equipment, International Research Center for Computational Mechanics, Department of Engineering Mechanics, Dalian University of Technology, 2 Linggong Rd, Dalian, Liaoning, 116024, China

[5]Department of Materials Science and Engineering, Northwestern University, 2145 Sheridan Road, Evanston, IL 60208, USA

[6]School of Physics and Electronics, Henan University, Jinming Rd, Kaifeng, Henan, 475004, China

[7]Flexterra Inc. 8025 Lamon Avenue, Skokie, IL 60077, USA

[⊥]B. Wang and A. Thukral contributed equally to this work.

[*]e-mail: xingeyu@cityu.edu.hk; cyu15@uh.edu; t-marks@northwestern.edu; a-facchetti@northwestern.edu.

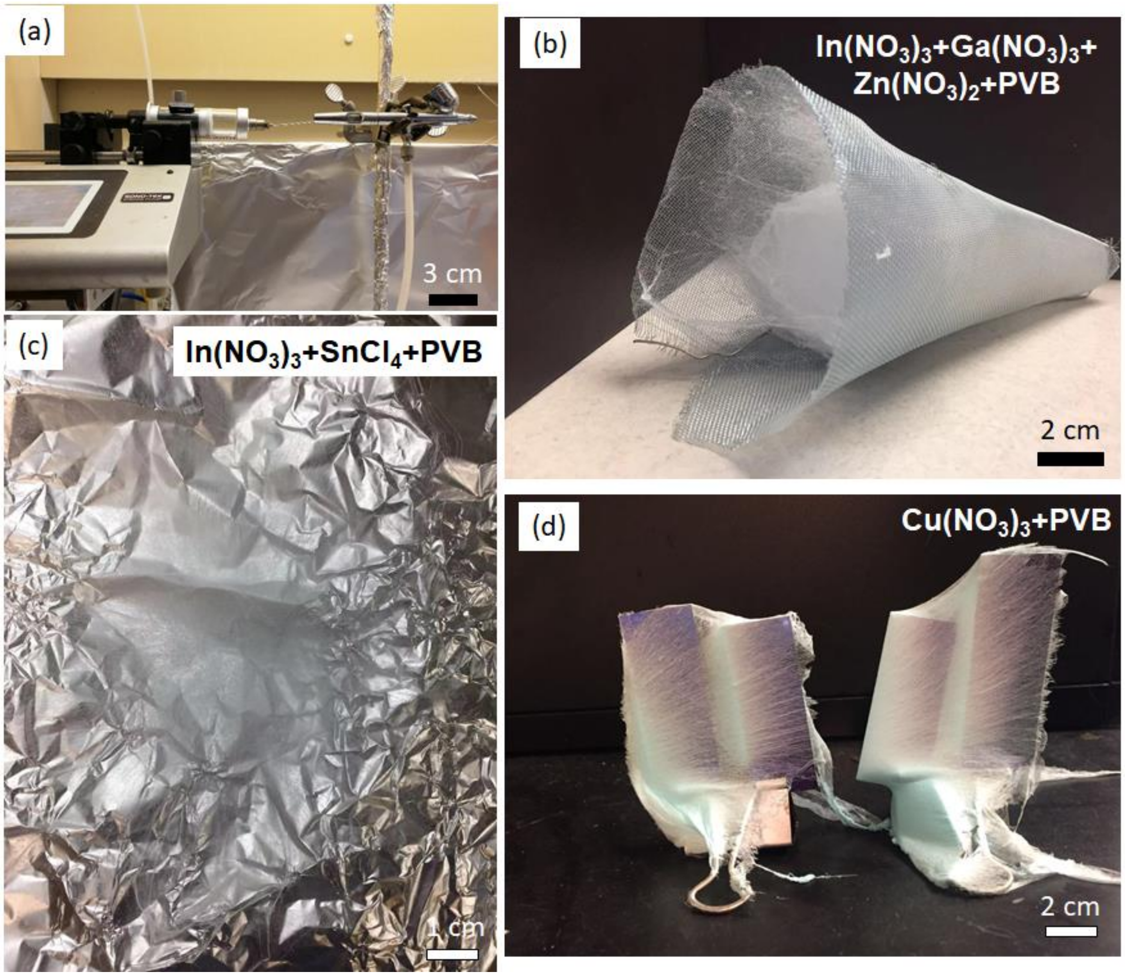

Supplementary Figure 1. (a) The setup of blow-spinning technique. Images of representative large-scale synthesis of polymer-based inorganic fibers by blow-spinning: (b) $In(NO_3)_3$, $Ga(NO_3)_3$ and $Zn(NO_3)_2$ in PVB fibers, (c) $In(NO_3)_3$ and $SnCl_4$ in PVB fibers, (d) $Cu(NO_3)_3$ in PVB fibers.

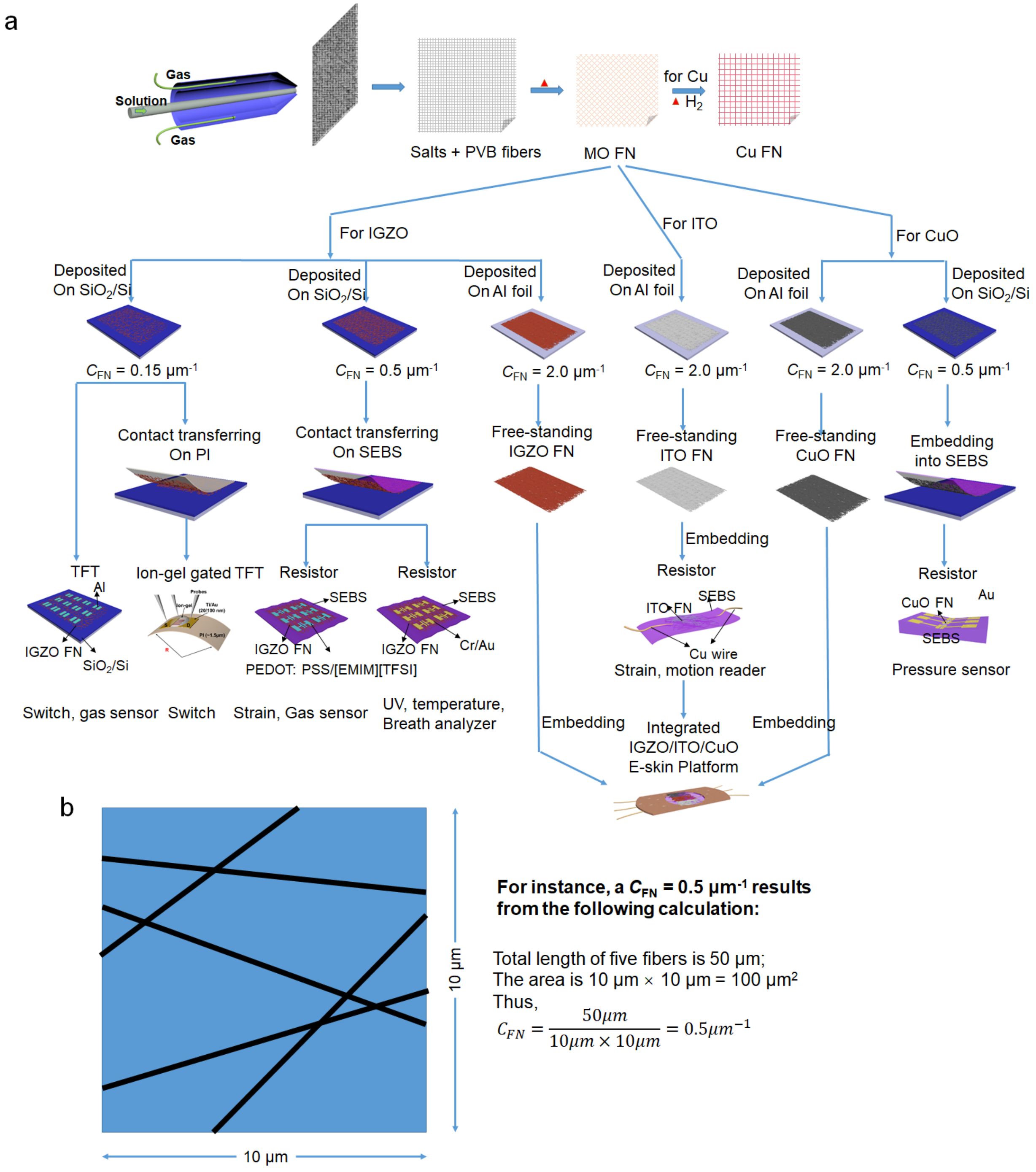

Supplementary Figure 2. (a) Fabrication process and device structures for all the devices discussed in this paper. (b) Schematic representation for defining the fiber coverage (C_{FN}) from supplementary reference 1. Note, for TFT applications, a low C_{FN} is optimum since additional excess fibers will prevent efficient switching. For resistor-based devices, middle/high C_{FN} is preferred since this enhances conductivity and, for sensors, sensitivity due to the high surface area. High-coverage fiber networks are optimum for self-standing conducting samples, which can be more easily integrated into monolithic devices.

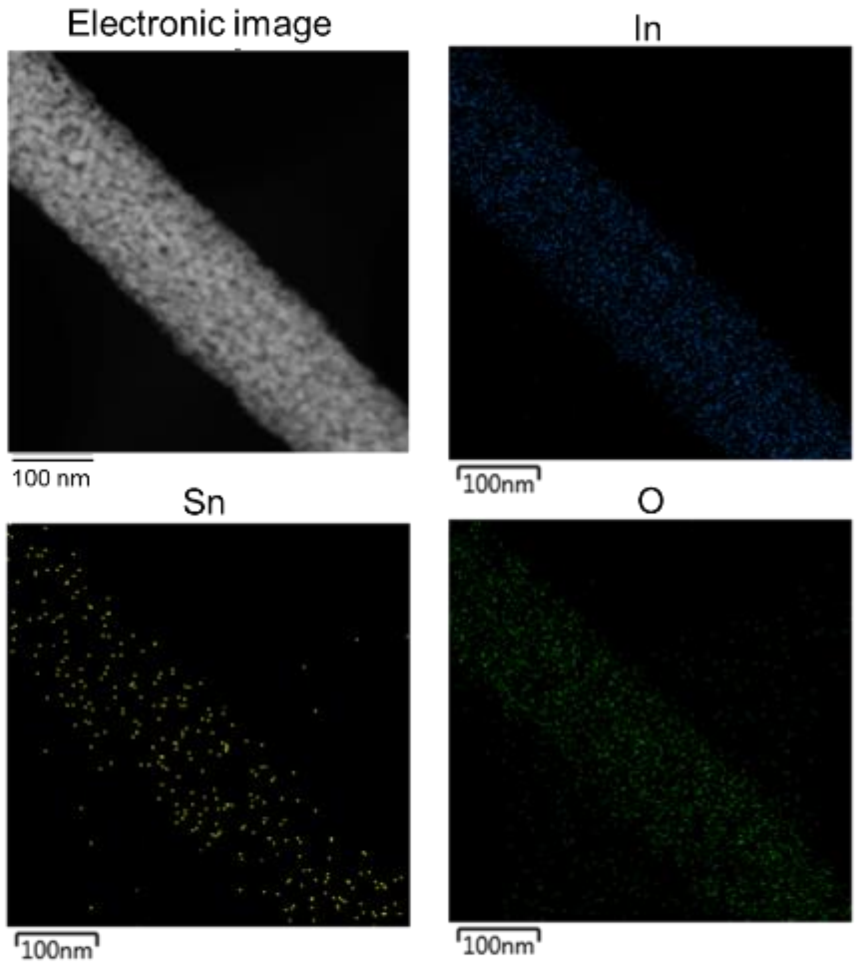

Supplementary Figure 3. Scanning transmission electron microscopy (STEM) of a single ITO fiber and Energy-dispersive X-ray spectroscopy scans for In, Sn, and O elements.

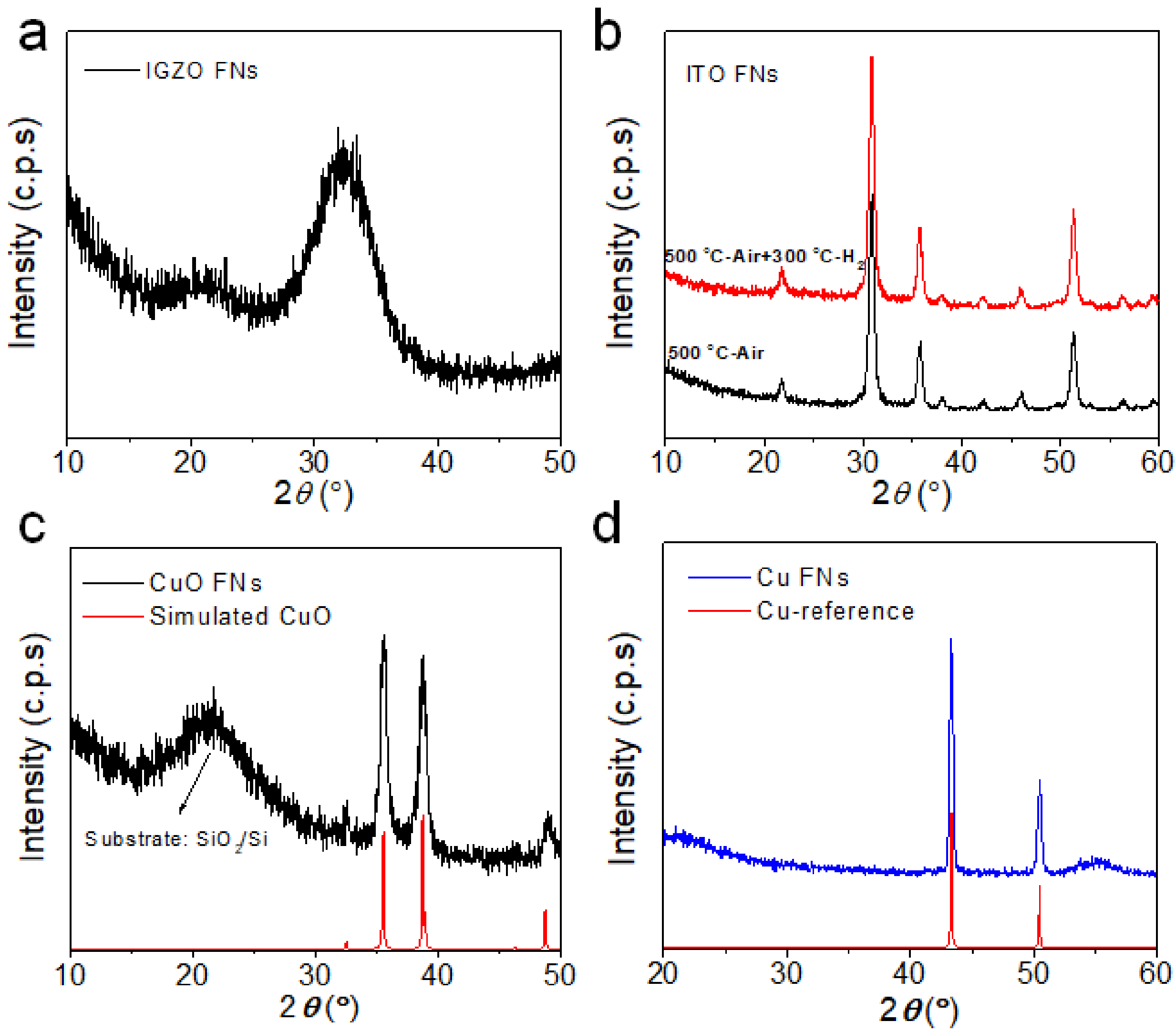

Supplementary Figure 4. GIXRD spectra of IGZO, ITO, CuO and Cu FNs (all C_{FN} = 0.5 μm^{-1}) on SiO_2/Si substrates.

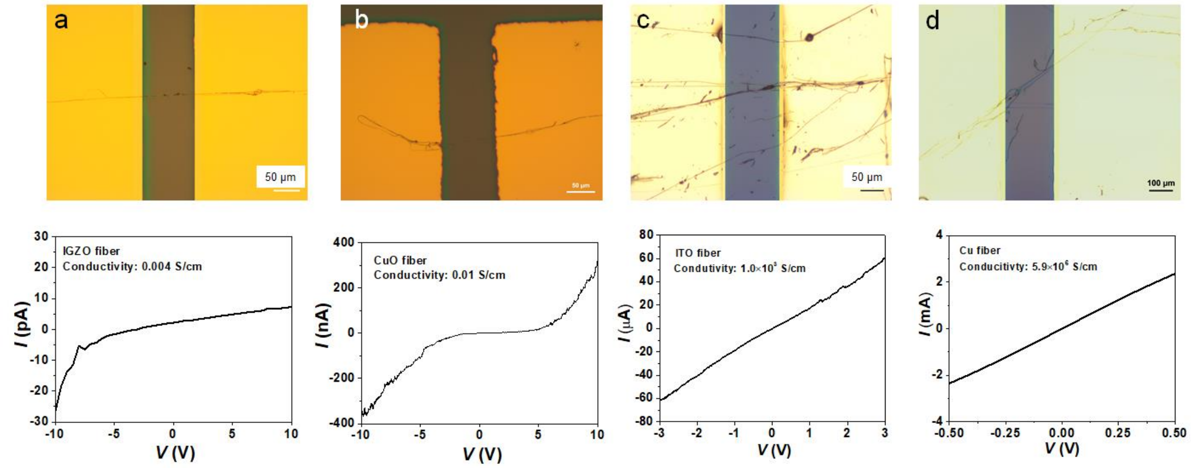

Supplementary Figure 5. Optical images and *I*-*V* curves for (a) IGZO, (b) CuO, (c) ITO and (d) Cu fibers. The fibers were fabricated on SiO_2/Si substrates. Evaporated 30 nm thick Au is used as electrodes with channel length of 100 µm. The conductivity of both fibers was evaluated by using following equation:

$$\sigma = \frac{L}{SR} = \frac{L}{n\pi r^2 R},$$

where L is channel length, n is fiber number, r is fiber radius, R is resistance obtained from *I*-*V* curves.

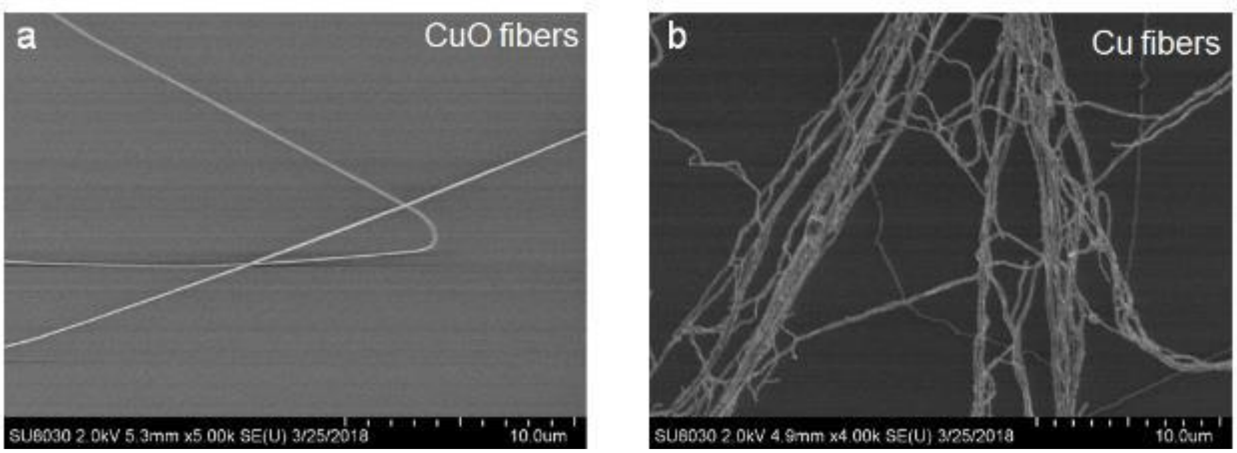

Supplementary Figure 6. SEM images of (a) CuO and (b) Cu fibers on SiO_2/Si substrates.

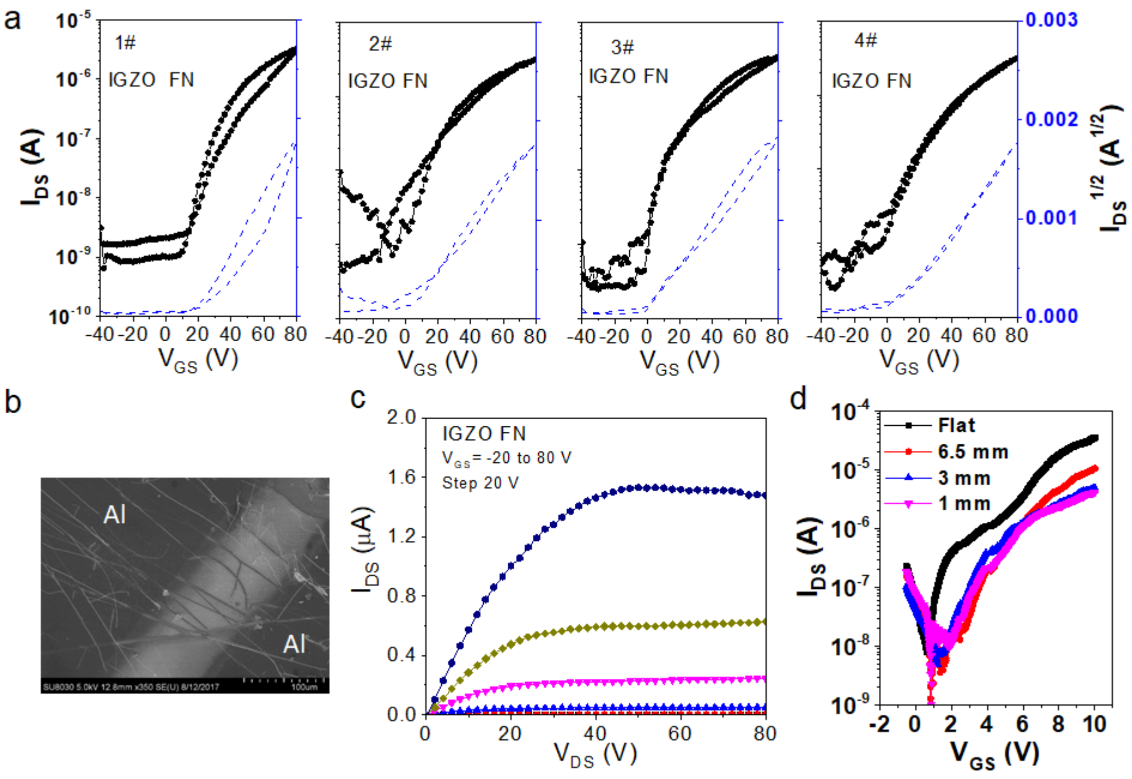

Supplementary Figure 7. (a) Transfer curves of IGZO FN-based TFTs on SiO_2/Si substrates from different batches, showing the good reproducibility. (b) SEM image of IGZO FN-based TFT with Al electrodes. (c) Representative output curve of an IGZO FN-based TFTs on SiO_2/Si substrates (C_{FN} = 0.15 μm^{-1}). (d) Transfer curves of flexible IGZO FN-based TFTs with ion-gel dielectric under the indicated bending radii.

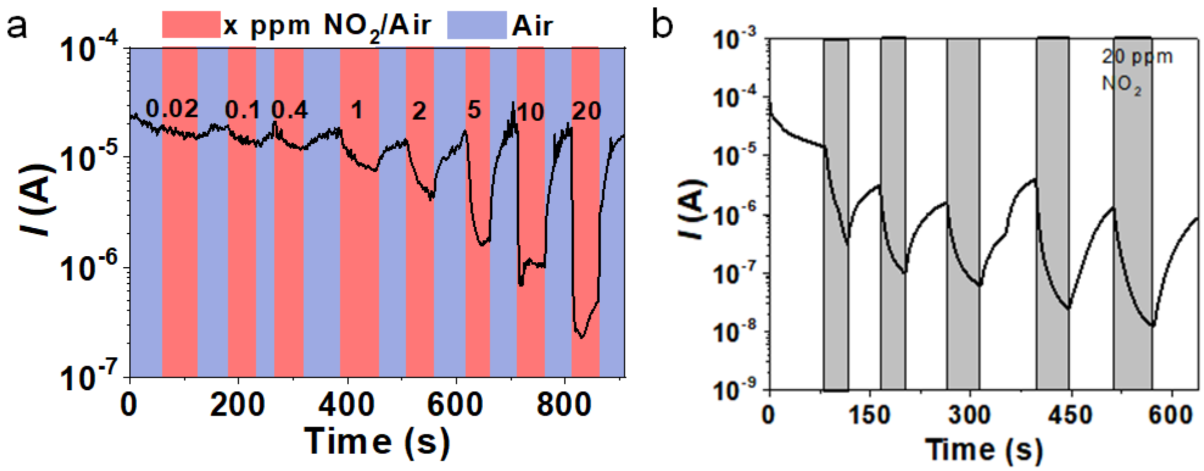

Supplementary Figure 8. (a) Real-time source-drain current (V_D=V_G=50 V) response to dynamic switching between NO_2 concentrations (20 ppb-20 ppm). The data are acquired from different batches, showing the good reproducibility. (b) Control TFTs as NO_2 sensors based on ~10 nm thick/dense IGZO film fabricated by solution combustion synthesis (from Ref 2). Both the gate and source-drain voltages are 80 V. Unlike the device based on IGZO FN, there is no current saturation when exposed to 20 ppm NO_2/Air. Thus, the response and recovery times are estimated to be >50 s and >60 s, respectively.

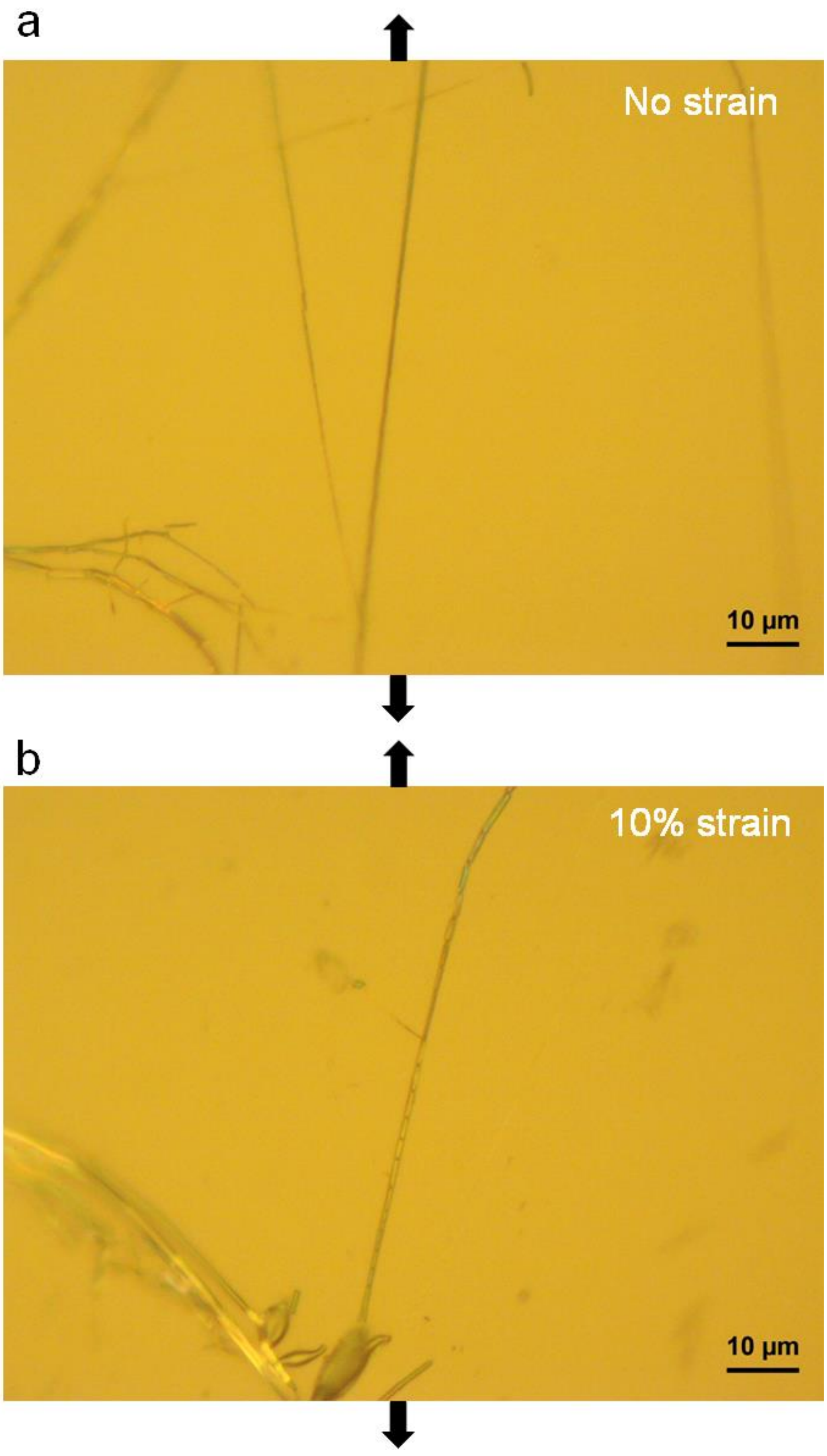

Supplementary Figure 9. Optical images of several IGZO fibers on a SEBS substrate under (a) no strain and (b) a 10% strain.

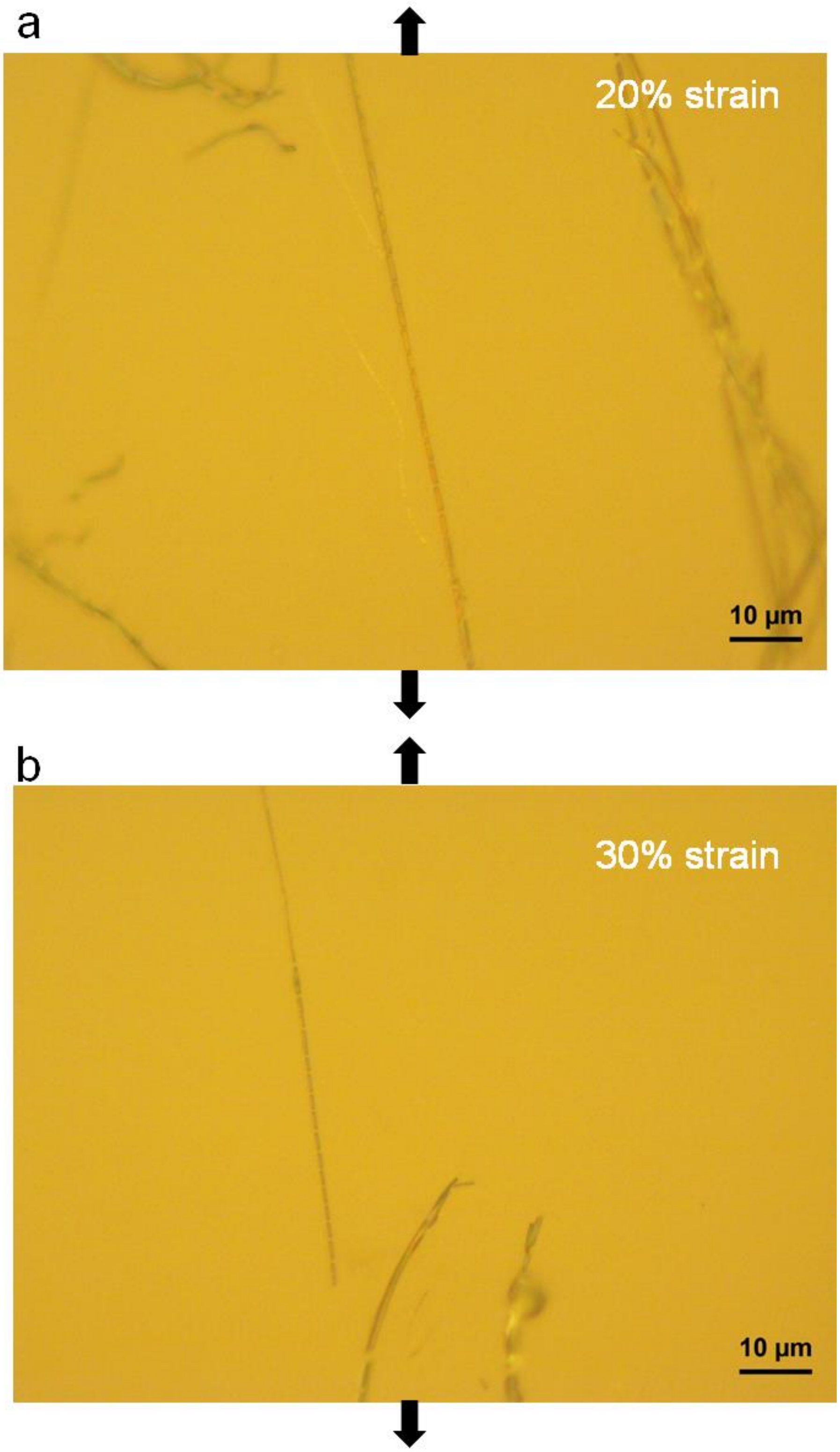

Supplementary Figure 10. Optical images of IGZO fibers on a SEBS substrate under (a) 20% and (b) a 30% strain.

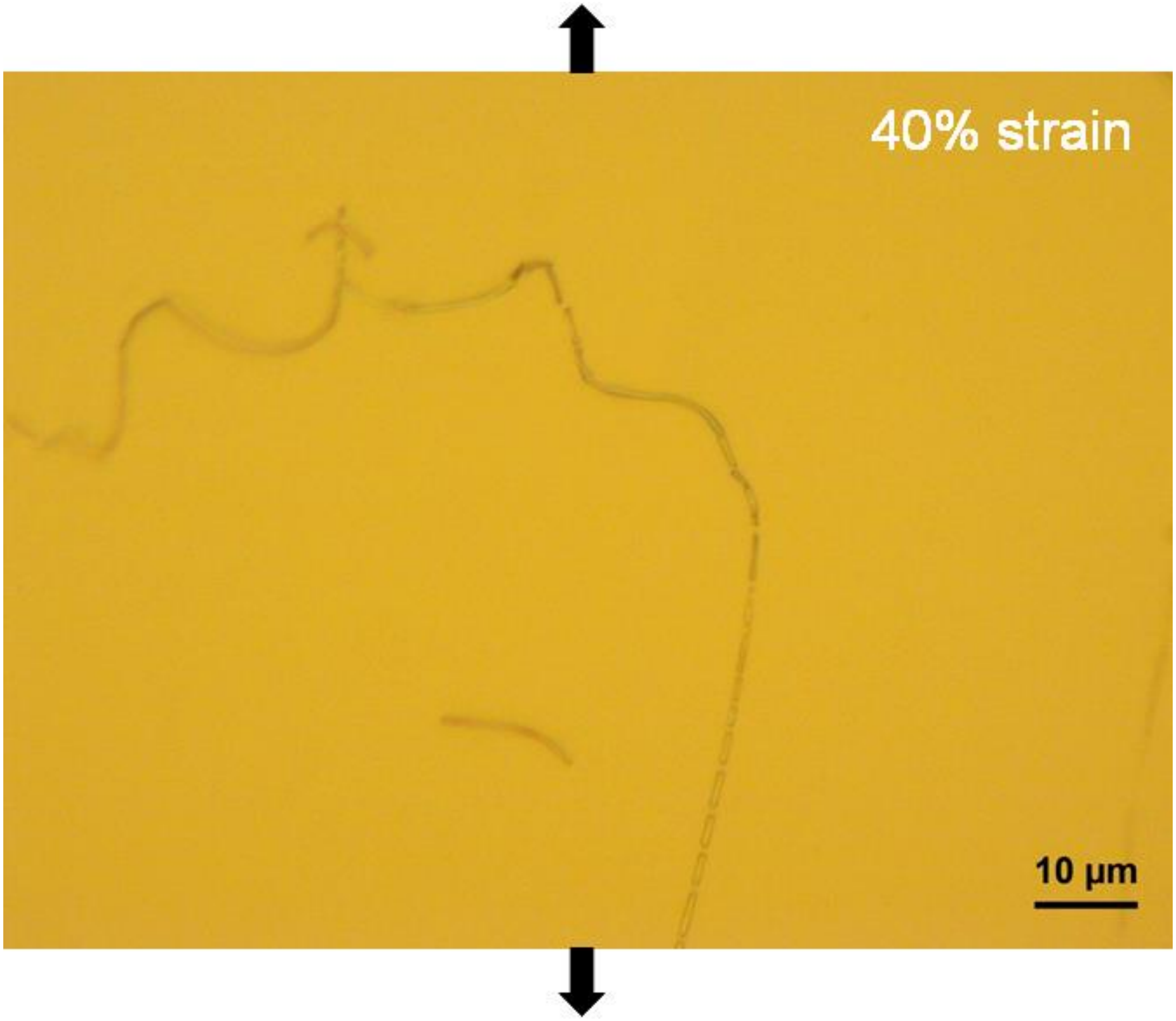

Supplementary Figure 11. Optical images of an IGZO fiber on a SEBS substrate under a 40% strain. The fiber section parallel to the stress direction exhibits several cracks while that perpendicular to the stress direction does not exhibit any crack.

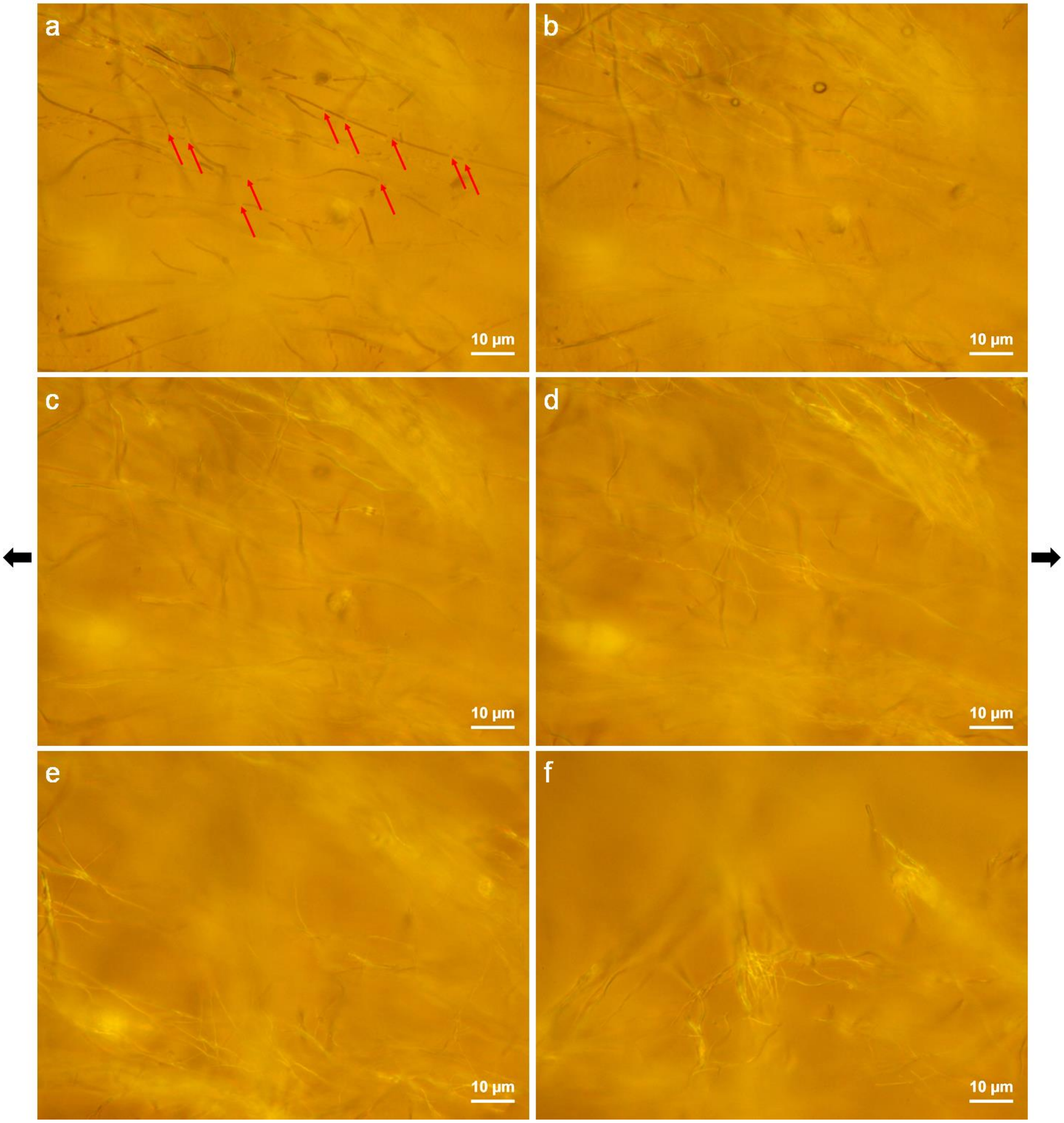

Supplementary Figure 12. Optical images of an IGZO NF (C_{FN} = 0.5 μm^{-1}) on a SEBS substrate under a 20% strain. The six images were recorded under different focuses, from the bottom to top of the film. Obvious broken fibers are observed for fibers contacting the SEBS substrate under 20% strain (S9a), while no breaks were recorded for the fibers located further away from the substrate.

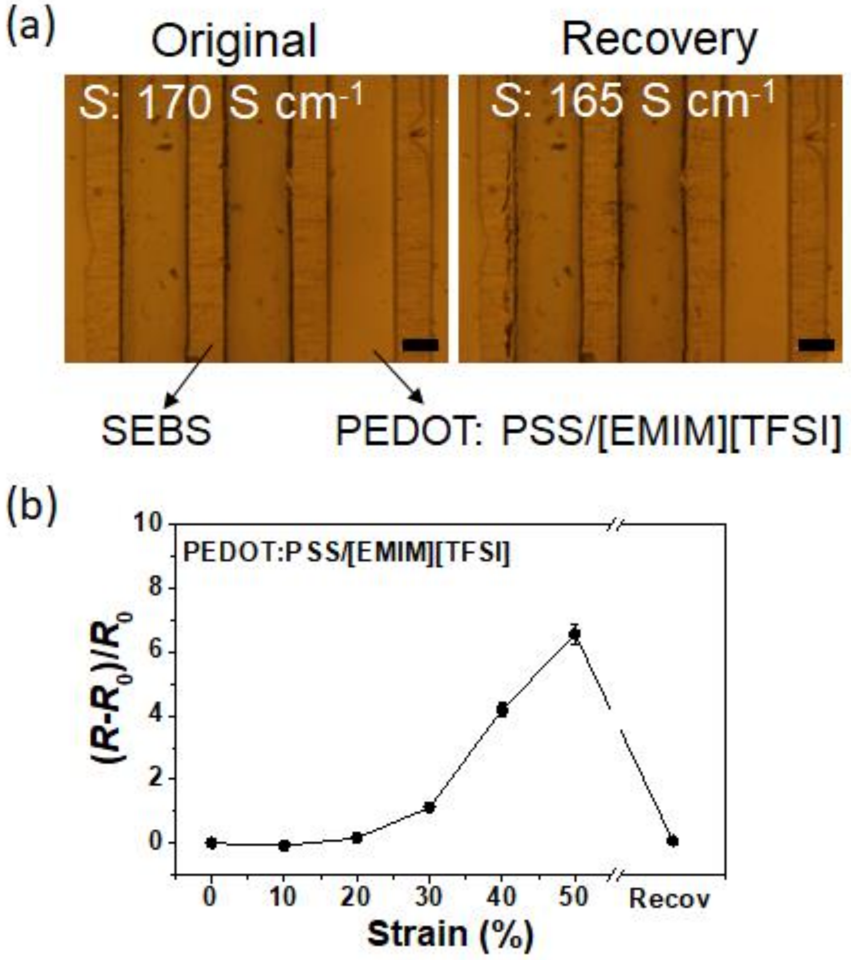

Supplementary Figure 13. (a) Optical images of a stretchable PEDOT: PSS/[EMIM][TFSI] electrode array before and after a 50% stretching. Scale bar is 100 µm. (b) Resistance change of the PEDOT: PSS/[EMIM][TFSI] electrode upon stretching. The initial conductivity of the PEDOT: PSS/[EMIM][TFSI] electrode is ~170 S cm^{-1} and it was tested for different tensional strains (0-50%). The relative resistivity change ($\triangle R/R_0$) first increases slowly to 0.16 when stretched to 20% then increases rapidly reaching ~7 when stretched to 50%. The conductivity returns close to the original upon relaxation without detectable damage of the electrodes.

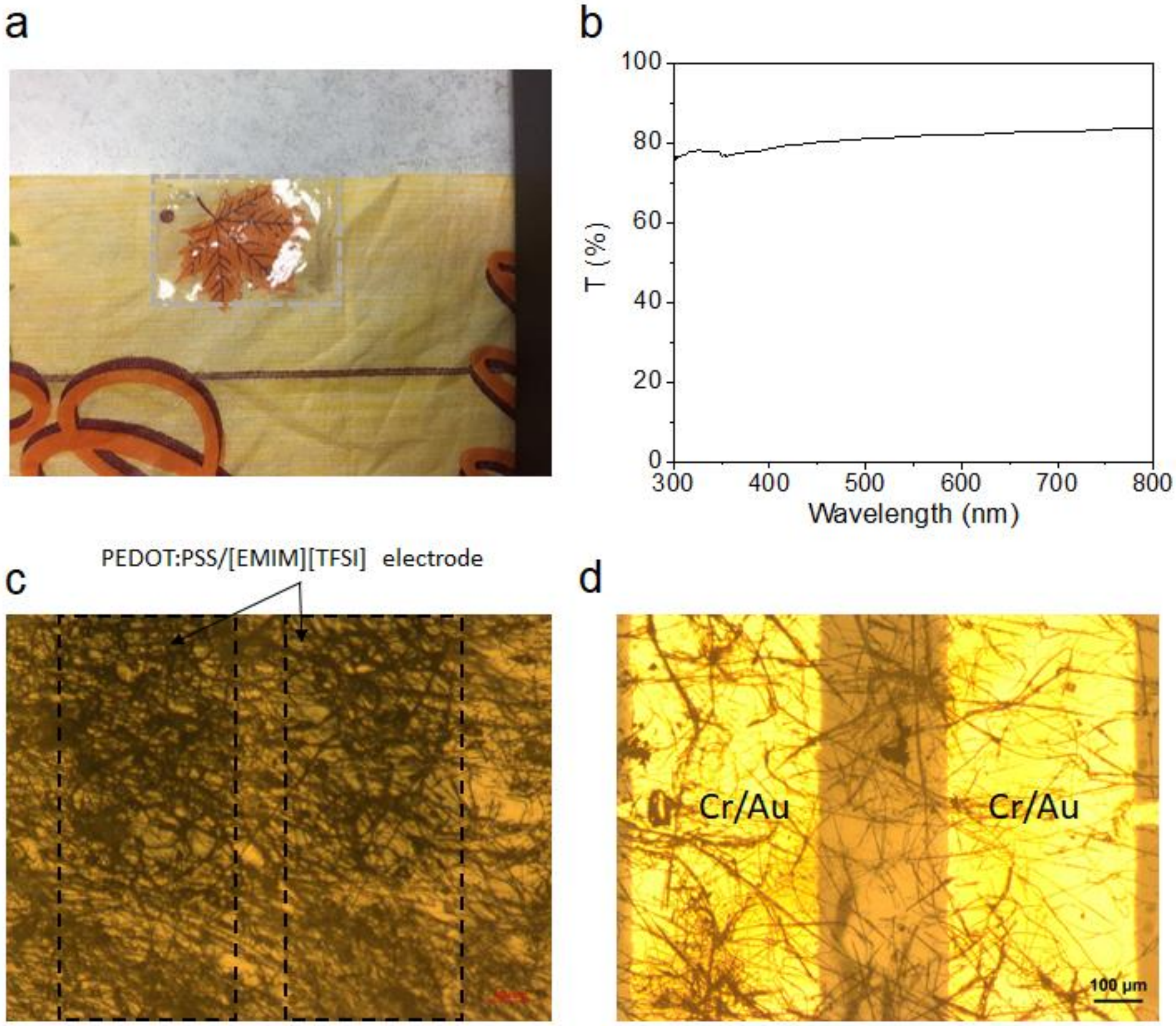

Supplementary Figure 14. (a) A photograph and (b) UV-Vis spectra of IGZO FN on SEBS substrate. Optical image of an IGZO FN (C_{FN} ~ 0.5 μm^{-1}) on a SEBS substrate with (c) PEDOT:PSS/[EMIM][TFSI] electrodes and (d) Au electrodes.

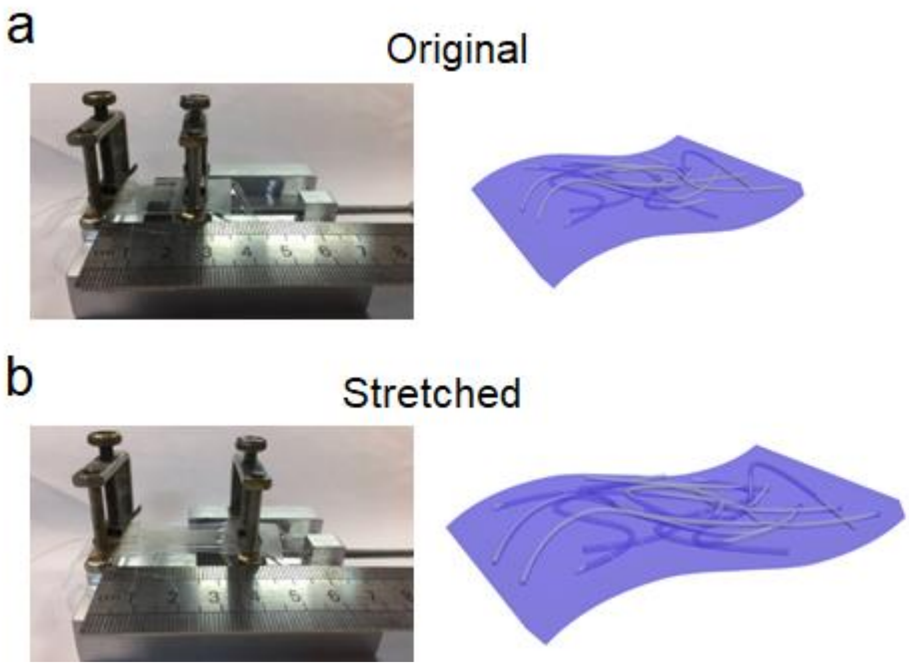

Supplementary Figure 15. Photographs and schematic morphologies of (a) pristine and (b) stretched IGZO FN (C_{FN} = 0.5 μm^{-1}) on SEBS substrates.

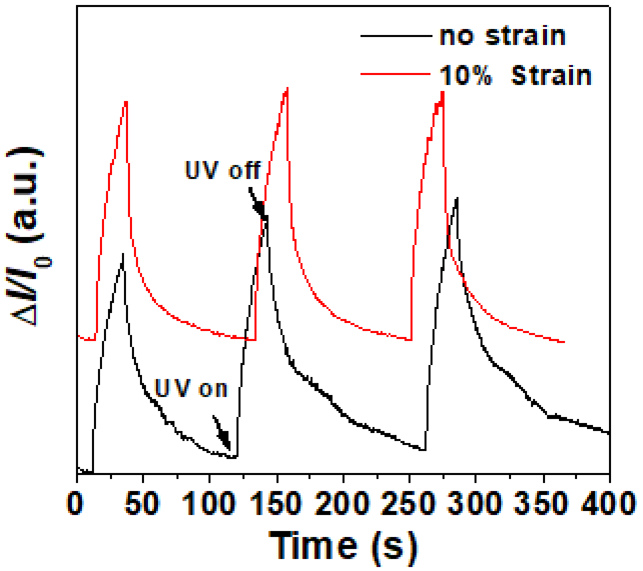

Supplementary Figure 16. Relative current change vs. time of an IGZO FN/SEBS device with/without UV irradiation (wavelength = 365 nm, power ~ 7.3 mW cm^{-2}). The C_{FN} is 0.5 µm^{-1} and the contacts are Cr(3 nm)/Au(50 nm). The device was tested without strain and upon a 10% strain, respectively.

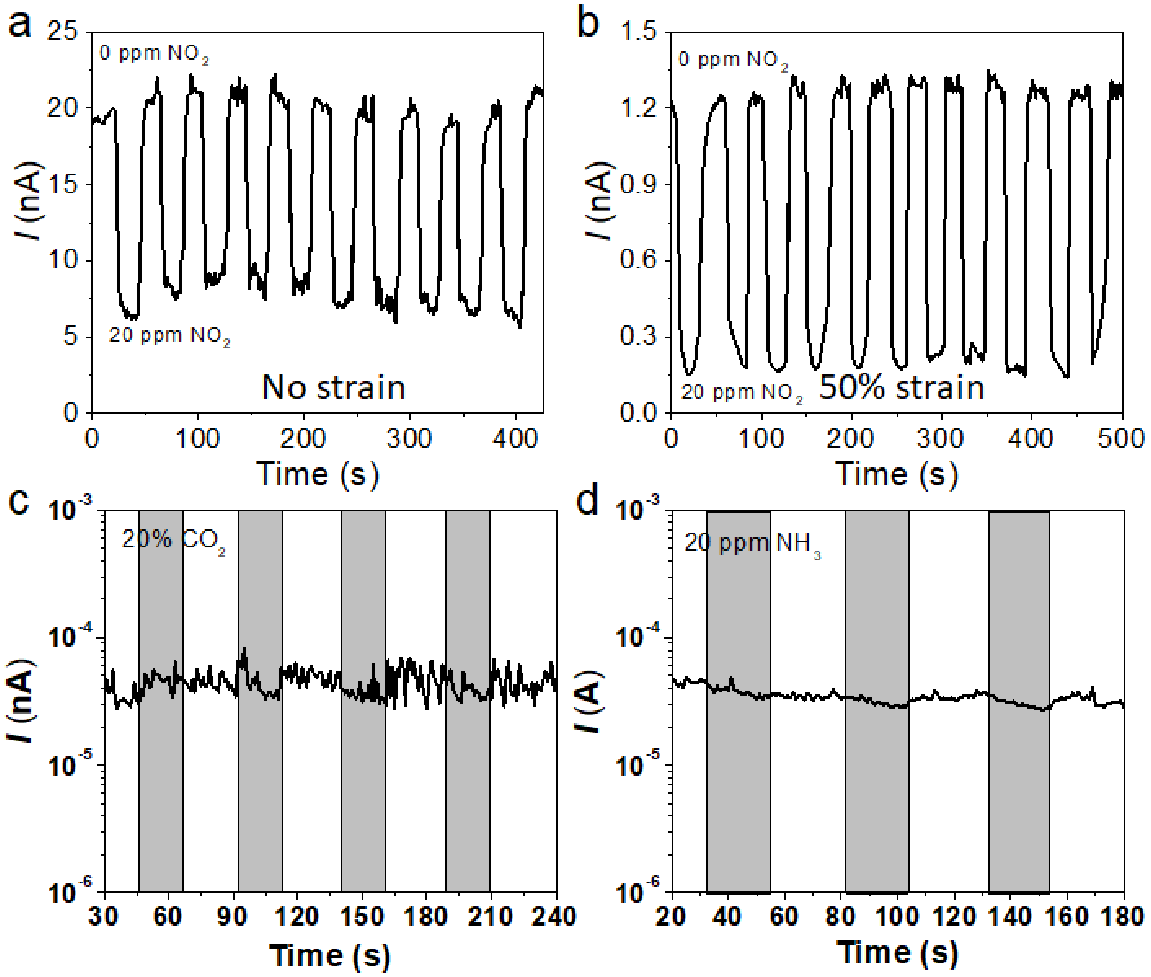

Supplementary Figure 17. (a) Cycling test for an IGZO FN/SEBS sensor from another batch exposed to 20 ppm NO_2 under no strain and 50% strain (bias = 5 V), showing the good reproducibility. *I*-*t* curves of a stretchable IGZO FN/SEBS device exposed to (a) 20% CO_2 and (b) 20 ppm NH_3. The C_{FN} is 0.5 μm^{-1} and the contacts are PEDOT:PSS/[EMIM][TFSI].

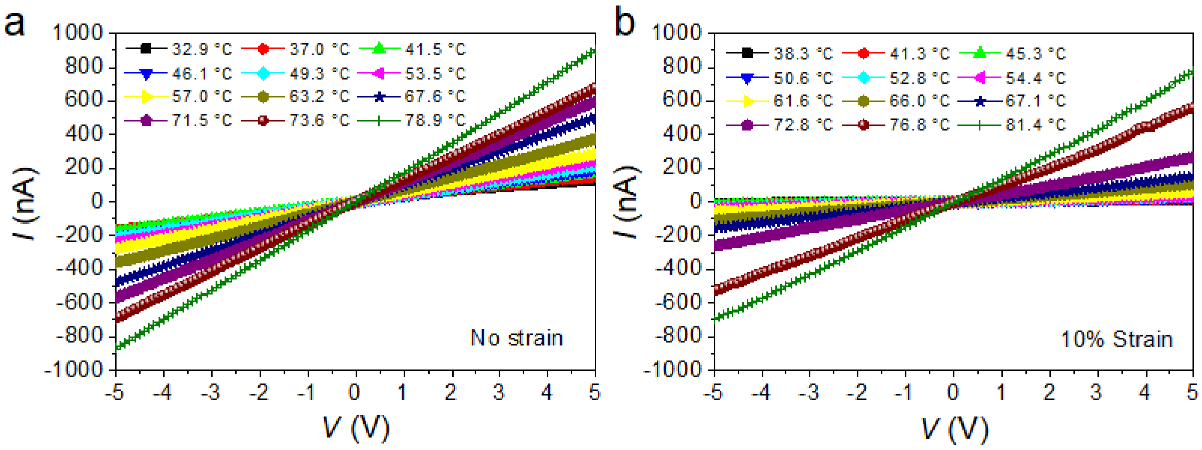

Supplementary Figure 18. *I*-*V* curves of a stretchable IGZO FN/SEBS device at different temperatures (a) without strain and (b) with a 10% strain. The C_{FN} is 0.5 μm^{-1} and the contacts are Cr(3 nm)/Au(50 nm).

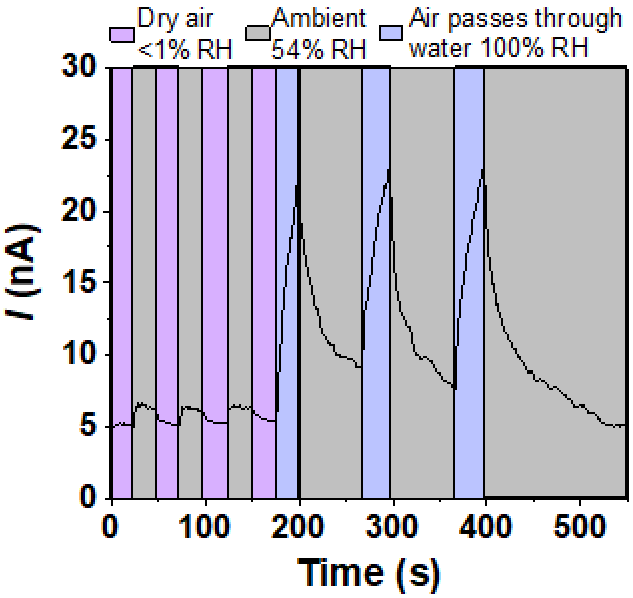

Supplementary Figure 19. Dynamic *I*-*t* curves of IGZO FN/SEBS device exposed to air with different humidity. The C_{FN} is 0.5 µm^{-1} and the contacts are Cr(3 nm)/Au(50 nm).

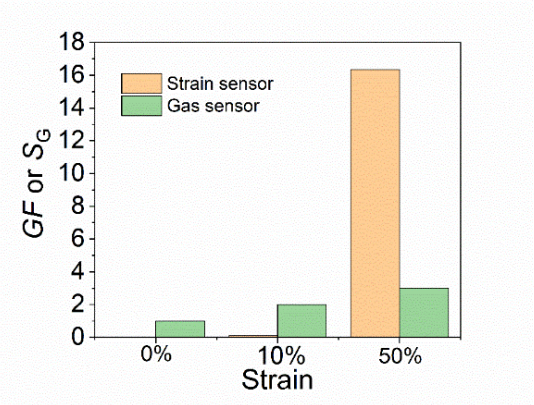

Supplementary Figure 20. Gauge factor and sensitivity to NO_2 for IGZO FN-based devices under different strain.

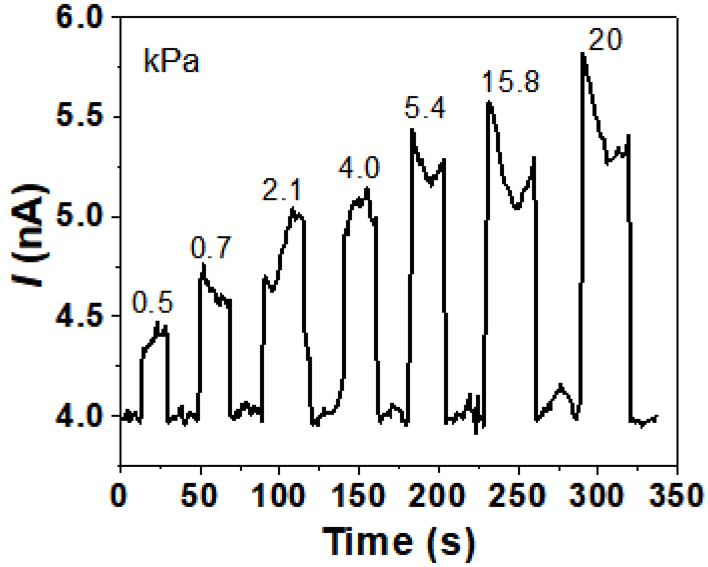

Supplementary Figure 21. *I-t* curves for a CuO FN/SEBS device with IGZO FN loaded under different pressures. The C_{FN} is 0.5 μm^{-1} and the contacts are Cr (3 nm)/Au (50 nm).

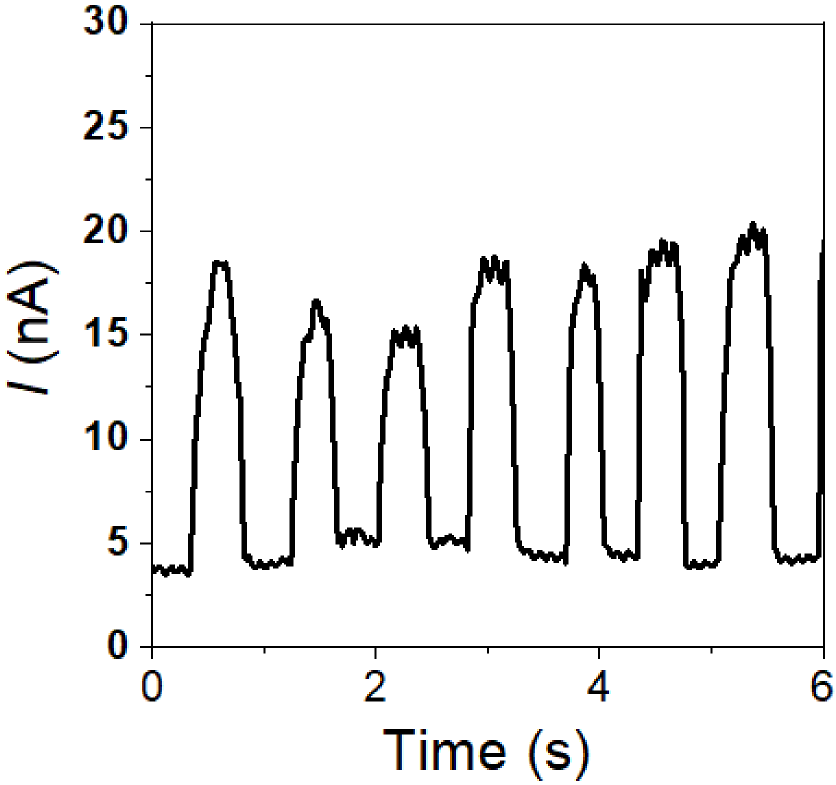

Supplementary Figure 22. *I-t* curves for a CuO FN/SEBS pressure sensor pressed by a finger for 7 times. The C_{FN} is 0.5 μm^{-1} and the contacts are Cr (3 nm)/Au (50 nm).

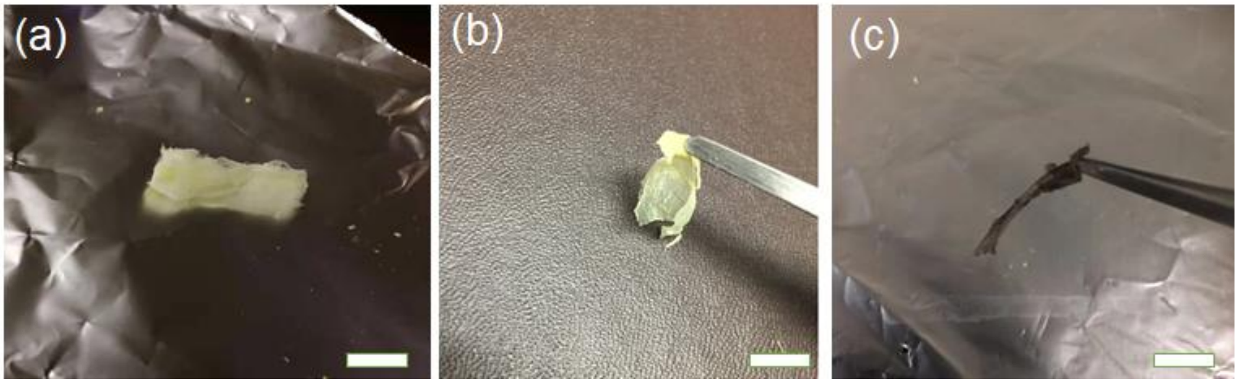

Supplementary Figure 23. Photograph of a free-standing (a) IGZO FN, (b) ITO FN, and (c) CuO FN. The C_{FN} is ~ 2.0 μm^{-1}.

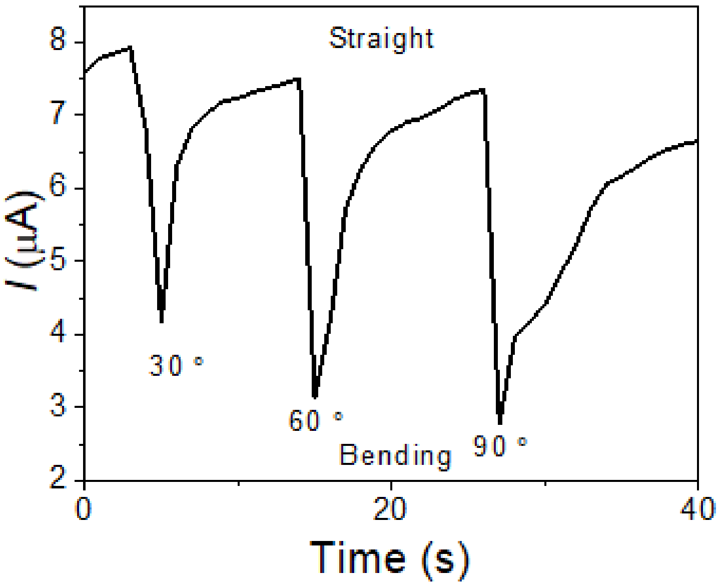

Supplementary Figure 24. *I-t* curves ITO FN/SBES device mounted on a finger when the finger bends at different degree.

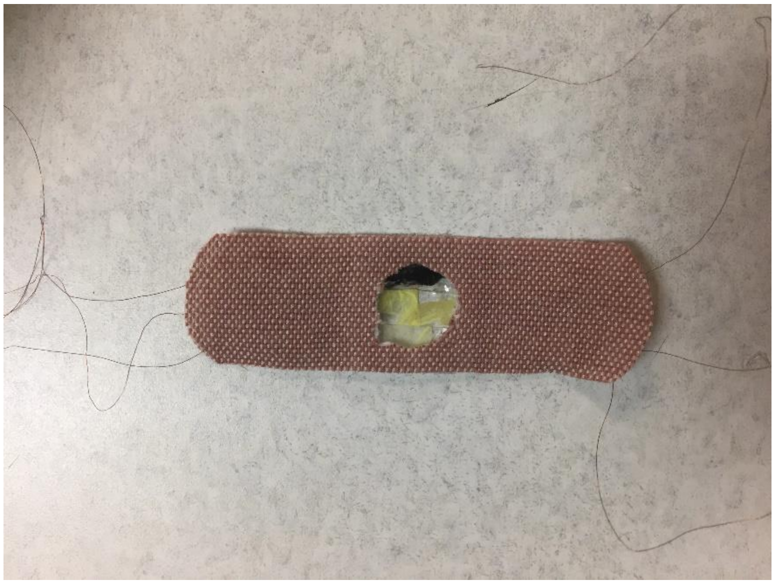

Supplementary Figure 25. Photograph of an integrated wearable device.

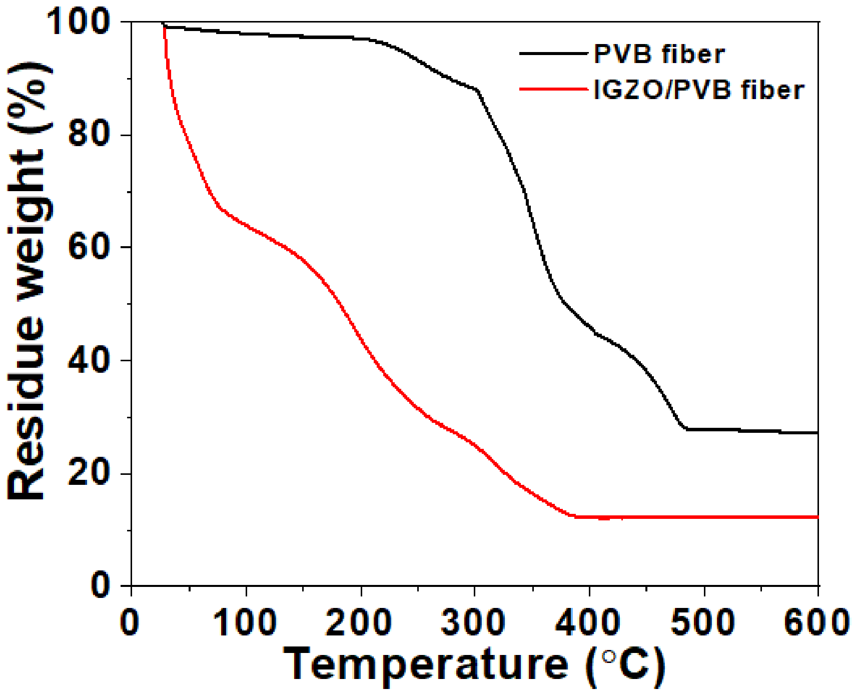

Supplementary Figure 26. TGA curves of PVB fiber and IGZO/PVB fiber. The heating rate is 10 °C/min and the dry air is used as the purging gas.

Supplementary Table 1. Comparison of the IGZO FN-based NO_2 gas sensor with literature reports.

**Material**	**Structure**	**Gas**	**Working conditions**	**LOD**	**Sensitivity or response**	**Response time**	**Year** [ref]
**Si**	Nanowires	NO_2	RT	500 ppb	<0.7%	30-40 s	2009[2]
**Te**	Nanofibers	NO_2	RT	10 ppm	0.6	1.4 min	2013[3]
**SnO_x**	Nanowires	NO_2	RT	9.7 ppm	89.2%	6 s	2012[4]
**WO_3**	Nanorods	NO_2	RT	1 ppm	1.7	40 s	2014[5]
**SnO_2/ZnO**	Nanowires	NO_2	UV illumination	1 ppm	238.7%	<20 s	2013[6]
**CuPc[a]**	film	NO_2	RT	415 ppb	60%/ppm	>10 min	2017[7]
**PANI[b]**	fibers	NO_2	RT	1 ppm	80%	50-60 s	2014[8]
**TIPS-pentacene[c]**	film	NO_2	RT	20 ppb	1000%/ppm	200 s	2017[9]
**PPy/NiO[d]**	composite	NO_2	RT	100 ppm	47%	49 s	2014[10]
**SWCNTs[e]**	nanotubes	NO_2	RT	6 ppm	0.034	-	2003[11]
**Graphene**	Nanosheet	NO_2	RT	5 ppm	27%	<90 s	2009[12]
**CNTs/SnO_2**	Nanowires	NO_2	RT	100 ppm	1.8	4.5 min	2009[13]
**Graphene/Cu_xO**	Nanosheet	NO_2	RT	97 ppm	95.1%	9.6 s	2014[14]
**ZnO@ZIF-CoZn[f]**	Nanowires	Acetone	260 °C	2 ppb	27	43 s	2016[15]
**IGZO**	Film	Ethanol	UV illumination	750 ppm	37%	53 s	2016[16]
**IGZO**	Film	NO_2	100 °C	5 ppm	200%/ppm	>2.5 min	2018[17]
**IGZO**	Film	NO2	UV illumination	< 2 ppm	3.47%/ppm	13.5 min	2018[18]
**IGZO**	Nanofibers	NO_2	RT	20 ppb	33.6%/ppm	2-3 s	This work

(a) CuPc: Copper(II) phthalocyanine; (b) PANI: Polyaniline; (c) TIPS-pentacene: 6,13-Bis(triisopropylsilylethynyl)pentacene; (d) PPy: Polypyrrole; (e) SWCNTs: single-wall carbon nanotubes; (f) ZIF: Zeolitic Imidazolate Framework.

Supplementary Note 1

How do IGZO FN-based resistors distinguish strain, light, temperature, gas and humidity?

1. First, the resistance increases upon exposure to NO_2 gas or when strained, while it decreases upon ambient light exposure, by increasing temperature, and increasing the humidity.
2. With regard to NO_2 gas and strain stimulus, we plotted the gauge factor (*GF*) and gas sensitivity (S_G) of IGZO FN-based devices under 0%, 10% and 50% strain below (Supplementary Figure 20). The *GF* is much smaller than S_G at 10% strain, indicating the NO_2 gas affects the resistance of devices under small strain. When the strain is 50%, we observe an opposite trend. Thus we can easily distinguish strain from NO_2 gas. Note, in actual use for wearable devices, the skin strain from the most body parts (such as fingers, forehead, etc.) is less than 10%, and thus the strain within such limit has negligible effect on the NO_2 gas detection.
3. In terms of light, temperature, and humidity, the sensitivity is ~16 mA W^{-1}, ~2.1%/°C and ~2%/%RH. The device is very sensitive to UV light, thus it can be distinguished easily. The differentiation of temperature and humidity requires the integrated e-skin platform.

Supplementary References

1. Meng Y., Liu G., Liu A., Guo Z., Sun W., Shan F. Photochemical activation of electrospun In_2O_3 nanofibers for high-performance electronic devices. *ACS Appl Mater Interfaces* **9**, 10805-10812 (2017).
2. Peng K. Q., Wang X., Lee S. T. Gas sensing properties of single crystalline porous silicon nanowires. *Appl Phys Lett* **95**, 243112 (2009).
3. Park H., Jung H., Zhang M. L., Chang C. H., Ndifor-Angwafor N. G., Choa Y., *et al.* Branched tellurium hollow nanofibers by galvanic displacement reaction and their sensing performance toward nitrogen dioxide. *Nanoscale* **5**, 3058-3062 (2013).
4. Jiang C., Zhang G., Wu Y., Li L., Shi K. Y. Facile synthesis of SnO_2 nanocrystalline tubes by electrospinning and their fast response and high sensitivity to nox at room temperature. *Crystengcomm* **14**, 2739-2747 (2012).
5. Wu Y. Q., Hu M., Wei X. Y. A study of transition from n- to p-type based on hexagonal WO_3 nanorods sensor. *Chinese Phys B* **23**, 040704 (2014).
6. Park S., An S., Mun Y., Lee C. Uv-enhanced NO_2 gas sensing properties of SnO_2-core/zno-shell nanowires at room temperature. *ACS Appl Mater Interfaces* **5**, 4285-4292 (2013).
7. Huang W., Zhuang X. M., Melkonyan F. S., Wang B. H., Zeng L., Wang G., *et al.* UV-ozone interfacial modification in organic transistors for high-sensitivity NO_2 detection. *Adv Mater* **29**, 1701706 (2017).
8. Zhang Y. X., Kim J. J., Chen D., Tuller H. L., Rutledge G. C. Electrospun polyaniline fibers as highly sensitive room temperature chemiresistive sensors for ammonia and nitrogen dioxide gases. *Adv Funct Mater* **24**, 4005-4014 (2014).
9. Wang Z., Huang L. Z., Zhu X. F., Zhou X., Chi L. F. An ultrasensitive organic semiconductor NO_2 sensor based on crystalline tips-pentacene films. *Adv Mater* **29**, 1703192 (2017).
10. Nalage S. R., Mane A. T., Pawar R. C., Lee C. S., Patil V. B. Polypyrrole-NiO hybrid nanocomposite films: Highly selective, sensitive, and reproducible NO_2 sensors. *Ionics* **20**, 1607-1616 (2014).
11. Li J., Lu Y. J., Ye Q., Cinke M., Han J., Meyyappan M. Carbon nanotube sensors for gas and organic vapor detection. *Nano Lett* **3**, 929-933 (2003).
12. Fowler J. D., Allen M. J., Tung V. C., Yang Y., Kaner R. B., Weiller B. H. Practical chemical sensors from chemically derived graphene. *ACS Nano* **3**, 301-306 (2009).
13. Lu G. H., Ocola L. E., Chen J. H. Room-temperature gas sensing based on electron transfer between discrete tin oxide nanocrystals and multiwalled carbon nanotubes. *Adv Mater* **21**, 2487 (2009).
14. Yang Y., Tian C. G., Wang J. C., Sun L., Shi K. Y., Zhou W., *et al.* Facile synthesis of novel 3D nanoflower-like cuxo/multilayer graphene composites for room temperature NO_x gas sensor application. *Nanoscale* **6**, 7369-7378 (2014).
15. Yao M. S., Tang W. X., Wang G. E., Nath B., Xu G. Mof thin film-coated metal oxide nanowire array: Significantly improved chemiresistor sensor performance. *Adv Mater* **28**, 5229 (2016).
16. Jaisutti R., Kim J., Park S. K., Kim Y. H. Low-temperature photochemically activated amorphous indium-gallium-zinc oxide for highly stable room-temperature gas sensors. *ACS Appl Mater Interfaces* **8**, 20192-20199 (2016).
17. Kim K. S., Ahn C. H., Jung S. H., Cho S. W., Cho H. K. Toward adequate operation of amorphous oxide thin-film transistors for low-concentration gas detection. *ACS Appl Mater Interfaces* **10**, 10185-10193 (2018).
18. Knobelspies S., Bierer B., Daus A., Takabayashi A., Salvatore G. A., Cantarella G., *et al.* Photo-induced room-temperature gas sensing with a-IGZO based thin-film transistors fabricated on flexible plastic foil. *Sensors-Basel* **18**, 358 (2018).
