# Supplementary material for: Flexible and stretchable metal oxide nanofiber networks for multimodal and monolithically integrated wearable electronics
Source: Nat Commun. 2020 May 15;11:2405. doi: 10.1038/s41467-020-16268-8 (PMC7229221; doi:10.1038/s41467-020-16268-8)
Supplement: Supplementary file 3 — Description of Additional Supplementary Files [file 41467_2020_16268_MOESM3_ESM.pdf]

Description of Additional Supplementary Files

Supplementary Movie 1: Fabrication of metal-oxide fibers by using blow-spinning method

Supplementary Movie 2: Current change of wearable ITO FN/SBES device upon bending.
